# Multiple burr hole and erythropoietin combination therapy: optimal early surgical intervention for patients with acute stroke episode of moyamoya disease or moyamoya syndrome

**DOI:** 10.3389/fneur.2024.1479379

**Published:** 2024-12-23

**Authors:** Yeonhu Lee, Jin Soo Lee, Seong-Joon Lee, Ji Man Hong, Yong Cheol Lim

**Affiliations:** ^1^Department of Neurosurgery, Ajou University Hospital, Ajou University School of Medicine, Suwon, Republic of Korea; ^2^Department of Neurology, Ajou University Hospital, Ajou University School of Medicine, Suwon, Republic of Korea

**Keywords:** moyamoya disease, moyamoya syndrome, multiple burr hole, erythropoietin, surgical intervention

## Abstract

**Objective:**

The optimal timing of bypass surgery for patients with moyamoya disease (MMD) or moyamoya syndrome (MMS) following an acute stroke episode remains unclear, mainly owing to the risk of postoperative complications. In this study, we aim to validate the safety and efficacy of early intervention using multiple burr hole (MBH) and erythropoietin (EPO) therapy, thereby refining the management strategy for patients with acute stroke episode of MMD or MMS.

**Methods:**

We retrospectively analyzed data from 70 patients with MMD or MMS who underwent MBH and EPO therapy. The cohort was divided based on the time interval between the latest neurological deterioration and surgery: early (<30 days) and later (≥30 days) groups. We evaluated and compared perioperative clinical parameters and the extent of neovascularization on a 6-month postoperative angiography. Long-term clinical outcomes, including transient ischemic attack (TIA), infarction, hemorrhage, and seizure, were also analyzed during the follow-up period.

**Results:**

In the cohort, 36 patients (51.4%) were in the early group, whereas 34 (48.6%) were in the later group. The 6-month follow-up angiography demonstrated that 34/47 hemispheres (72.3%) in the early group exhibited successful neovascularization (≥2/3 of MCA territories) compared with the 19/44 (43.2%) hemispheres in the later group (odds ratio [OR] = 3.44; 95% confidence interval [CI]: 1.46–8.45; *p* < 0.01). In addition, a notable reduction (≥50%) in basal moyamoya vessels was observed in 30/47 hemispheres (63.8%) from the early group vs. 12/44 (27.3%) hemispheres from the later group (OR = 4.71; 95% CI: 1.97–11.82; *p* < 0.001). During the average follow-up of 56.5 months, only six patients experienced infarction or hemorrhage.

**Conclusion:**

Our dataset suggests that MBH and EPO combination therapy is an effective, minimally invasive, and acceptable treatment, even in the early period of patients with MMD or MMS following an acute stroke episode.

## 1 Introduction

Moyamoya disease (MMD) is a progressive angiopathy characterized by stenosis of the supraclinoid internal carotid arteries (ICAs) and the formation of abnormal moyamoya vessels. As the disease progresses to Suzuki stages 5–6, a notable reduction is observed in these moyamoya vessels with increased collaterals, decreasing the risk of ischemia or hemorrhage (1, 2).

Bypass surgery is an essential therapeutic modality for patients with MMD and moyamoya syndrome (MMS), facilitating a safer and expedited transition to Suzuki stages 5–6 and diminishing the long-term risk of stroke (3–6). Notably, direct and indirect bypass surgeries yield beneficial results; however, several studies have reported risks of postoperative stroke and neurological deterioration for patients with acute stroke episode of MMD or MMS. These are attributed mainly to the inherent risks associated with general anesthesia and potential for intraoperative hemorrhage (7–12). Although the risk rates vary across studies, patients undergoing surgical bypass within 90 days from the presentation of acute stroke have been reported to experience postoperative stroke and neurological deterioration in 15–22% and 17–33% of cases, respectively (13, 14).

Notably, during these acute periods, patients most urgently require transdural collaterals and blood flow enhancement, which can be facilitated through bypass surgery. Recognizing this challenge, we have explored the feasibility of using the multiple burr hole (MBH) under local anesthesia as a minimally invasive surgical technique for these high-risk patients (15). In addition, we have also been investigating the potential of using erythropoietin (EPO) as an adjunctive agent in promoting neovascularization (16, 17).

MBH and EPO therapy have been utilized as our institutional strategy to reduce invasiveness and enhance neovascularization. Therefore, in this study, we aimed to ascertain the optimal type and timing of bypass surgery for patients with an acute stroke episode of MMD or MMS. We also compared the clinical and angiographic outcomes between patients who underwent surgery early vs. those who underwent surgery later to investigate the appropriate timing of surgery.

## 2 Methods

### 2.1 Study design and patient’s selection criteria

In this retrospective study, we evaluated patients diagnosed with MMD or MMS who underwent MBH at a tertiary medical center between April 2011 and August 2022. The study included 70 patients who met the following inclusion criteria: (1) a confirmed diagnosis of MMD or MMS using digital subtraction angiography (DSA), following the guidelines established by the Research Committee on Moyamoya Disease of the Ministry of Health and Welfare of Japan; (2) age ≥ 18 years; (3) absence of previous bypass surgeries; (4) completion of a preoperative DSA within 3 months preoperatively, supplemented by at least one additional imaging test including perfusion computed tomography (CTP), perfusion magnetic resonance imaging (MRP), or single-photon emission computed tomography (SPECT); and (5) a follow-up DSA performed between 6 and 12 months postoperatively.

Data on outcomes were collected from the electronic medical records system. The Ajou University Medical Center’s Institutional Review Board approved this study (AJOUIRB-DB-2023-373).

### 2.2 Data collection and clinical courses

Demographic data included age, sex, smoking habits, moyamoya laterality, and the presence of comorbid conditions such as hypertension, diabetes mellitus, and dyslipidemia. A “smoker” was defined as an individual who has smoked at least 100 cigarettes in their lifetime and continues to smoke cigarettes at the time of the study. The National Institutes of Health Stroke Scale (NIHSS) and modified Rankin Scale (mRS) scores were measured at three different time points: admission, discharge, and 6 months postoperatively. Patients were categorized into the “early” or “later” groups based on their timing of surgery. The early group comprised patients who had undergone MBH <30 days from the most recent neurological event owing to transient ischemic attack (TIA), ischemic stroke, or hemorrhagic stroke. We defined a neurological events as the occurrence of any neurological symptoms solely related to moyamoya, not due to other causes. Conversely, the later group consisted of patients who had undergone MBH ≥30 days from the most recent neurological event. In a previous study analyzing perioperative stroke risk in patients with MMD, “recent stroke” was defined as occurring within 6 weeks (11). However, in our study, we have adopted a narrower timeframe to assess the effects of early intervention more stringently. Patients in the later group mostly underwent surgeries scheduled during outpatient visits and were admitted as regular inpatients for the procedure. Presentation types were categorized into three groups based on the most recent events: TIA, ischemic stroke, or hemorrhagic stroke. During the pre-operative period, clinical courses were evaluated based on the NIHSS score at admission. When the NIHSS score increased by ≥2 from the value at admission, the clinical course was defined as either “fluctuation” or “progression.” “Fluctuation” was used when the score returned to the value at admission, whereas “progression” was used if the worsened state persisted. The same criteria, based on the NIHSS score at the time of surgery, were applied to evaluate the postoperative clinical course. When there was a ≥ 2 decrease in the NIHSS score from the value at admission, the clinical course was defined as “improvement.” We also assessed the clinical outcomes during the follow-up period, including the incidence and timing of TIA, ischemic stroke, hemorrhagic stroke, and seizure.

### 2.3 Hemisphere analysis

**Figure 1:**
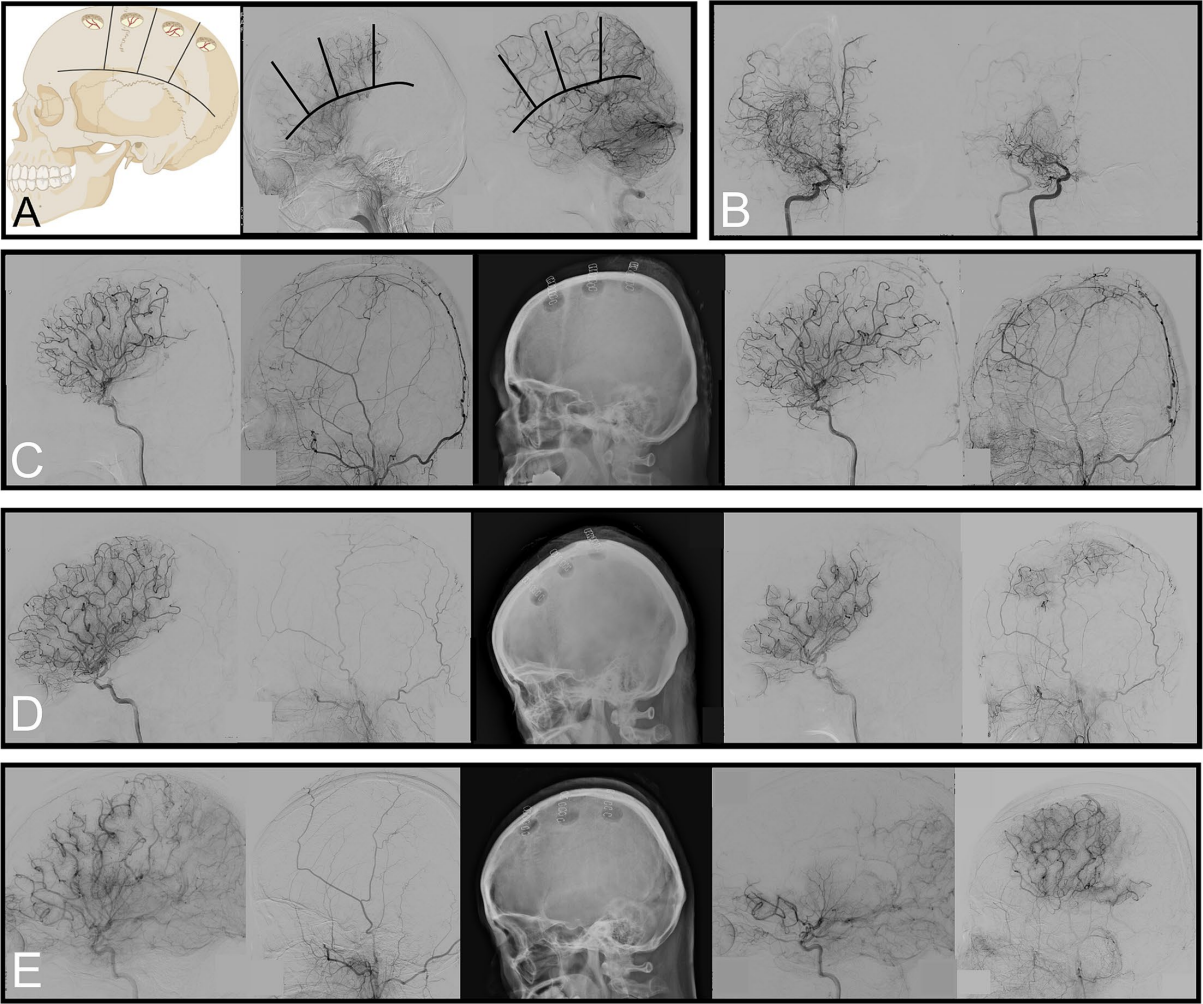
Criteria to evaluate regional vascularity, reduction of basal moyamoya vessels, and scales used to measure neovascularization. **(A)** Examples of “impaired” vascularity in the frontal and coronal suture regions and “not impaired” vascularity in the parietal and posterior parietal regions. **(B)** Angiography showing ≥50% of basal moyamoya vessels 6 months after surgery. **(C–E)** Preoperative angiography (left) and 6-month follow-up (right). Each of these is an example of Matsushima grades C, B, and A with regional neovascularization status, showing “no replacement,” “balanced,” and “replaced,” respectively.

In total, 121 hemispheres with moyamoya were assessed and divided into two groups: hemispheres that underwent MBH and hemispheres that did not. The angiographic stage was evaluated using the Suzuki staging system. Vascularity was assessed by examining the circulation of the ICA, external carotid artery (ECA), and vertebral artery (VA) in the lateral DSA view. If <66% of the hemisphere showed vascular supply in the capillary phase, the vascularity was classified as “impaired” (Supplementary Figure 1A); otherwise, it was classified as “not impaired” (Supplementary Figure 1B). The vascularity of each region where the burr hole was created was evaluated by dividing it into the frontal, coronal suture, parietal, and posterior parietal regions. The same criteria of “not impaired” and “impaired” were used to evaluate regional vascularity (Figure 1A). Preoperative regional perfusion status was assessed using CTP, MRP, or SPECT images. Regional perfusion impairment (RPI), acute infarction (detected using MR diffusion-weighted imaging), and chronic infarction (no restriction on MR diffusion-weighted imaging but with hypodensity on CT) were evaluated in the anterior cerebral artery (ACA), middle cerebral artery (MCA), and posterior cerebral artery (PCA) territories.

Follow-up DSA was conducted at least 6 months postoperatively to reassess the angiographic characteristics. A subgroup analysis was performed on the 91 hemispheres that underwent surgery. We evaluated whether the basal moyamoya vessels originating from the ICA had decreased by ≥50% (Figure 1B). We assessed the Matsushima grade, a scale used to measure neovascularization after bypass surgery (grade A: proportion of the MCA territory with revascularization from collaterals from the ECA through the burr holes was more than two-thirds; grade B: between one-third and two-thirds; grade C: <one-third) (18). The degree of neovascularization was assessed for each region in which surgery was performed; if no burr hole was made, it was recorded as “not done.” If the existing vessels remained unchanged on DSA after burr hole surgery, they were classified as “no replacement.” If the pre-existing and new vessels formed through the burr hole contributed to the region’s blood supply, it was classified as “balanced.” If the pre-existing vessels vanished and most (≥66%) of the blood supply was delivered by the new vessels formed through the burr hole, it was classified as “replaced” (Figures 1C–E).

### 2.4 Surgical procedure

EPO premedication before MBH was performed as described in previous literature. Total 120,000 unit of Intravenous EPO (Epokine; CJ healthcare, South Korea) was injected for three consecutive days right before the surgery (15). In the operating theater, the patients were placed in a supine position. Local anesthesia was administered through a lidocaine injection at predetermined incision points. A 3–4-cm scalp incision was made for each burr hole, and the burr holes, each 2–3 cm in size, were drilled using the Midas Rex® high-speed drill (Medtronic, Minneapolis, Minnesota, United States). Any bone bleeding from the diploic veins was managed using bone wax. At each burr hole, a cruciate incision was placed in the dura to break the mechanical barrier for collateral formation. Subsequently, the scalp incisions were closed with 2–0 vicryl sutures and staples. Up to four burr holes were performed per hemisphere, specifically targeting regions with impaired vascularity or perfusion impairment as identified on preoperative imaging.

### 2.5 Statistical analysis

Statistical analyses were performed using R software (version 4.2.1; R Foundation for Statistical Computing, Vienna, Austria). The basic characteristics of each patient, hemisphere, and region were analyzed using the chi-square, Fisher’s exact, t-test, and Mann–Whitney U tests, respectively. Statistical significance was set at *p* < 0.05. Variables taken at multiple time points were analyzed using the Friedman test.

Logistic regression was used to identify factors that may have influenced the decision to perform surgery in each region and were associated with the level of neovascularization (“replaced,” mentioned above). Variables with a *p*-value less than 0.05 in the univariable analysis were included in the multivariable analysis. Hemispheric improvements were assessed based on two criteria: the achievement of a Matsushima grade A and a ≥ 50% decrease in basal moyamoya vessels from the ICA.

Survival analysis was conducted using clinical follow-up data to evaluate the occurrence and timing of events, such as TIA, infarction, hemorrhage, and seizure.

## 3 Results

### 3.1 Baseline characteristics and perioperative clinical outcomes

The 70 patients were divided into early (*n* = 36) and later (*n* = 34) groups. Baseline characteristics, including age, sex, and common comorbidities, were similar between groups. During the preoperative period, significantly more patients in the early group experienced neurological fluctuations or progression (*p* < 0.001). Postoperatively, 77.8% of patients in the early group showed no neurological worsening, with 10 patients showing improvement. Of the eight patients with postoperative neurological worsening, six recovered by discharge. The mean preoperative hospital stay was longer in the early group (*p* = 0.001), but no significant difference was observed postoperatively. Overall, 10.0% of patients had surgical site complications. Three patients (4.3%) experienced lesion extension, which is the progression of a pre-existing acute infarction (Table 1). Of these cases, two were incidental findings on follow-up imaging, not associated with neurological worsening; one involved an extension of the MCA territory infarction, neurologically recovered with medical treatment. The clinical details are described in Supplementary Table 1. In the Early group, the duration from the neurological event to surgery was a median of 12 days, with an interquartile range (IQR) of 3 days. The shortest was 3 days and the longest was 25 days. In the Later group, the median was 131 days, with an IQR of 61 days, the shortest being 41 days and the longest being 254 days.

**Table 1:** Baseline characteristics and perioperative courses of patients.

	All patients (*n* = 70)	Early group (*n* = 36)	Later group (*n* = 34)	*p*-value
Sex (Female)	46 (65.7)	28 (77.8)	18 (52.9)	0.053
Age (years)	43.4 (12.7)	43.2 (11.4)	43.7 (14.1)	0.875
HTN	26 (37.1)	13 (36.1)	13 (38.2)	0.999
DM	18 (25.7)	10 (27.8)	8 (23.5)	0.894
Dyslipidemia	14 (20.0)	10 (27.8)	4 (11.8)	0.169
Smoking	21 (30.0)	11 (30.6)	10 (29.4)	0.999
Moyamoya syndrome	2 (2.9)	1 (2.8)	1 (2.9)	0.999
Unilateral moyamoya	18 (25.7)	9 (25.0)	9 (26.5)	0.999
Presentation type				0.807
TIA	3 (4.3)	2 (5.6)	1 (2.9)	
Ischemic stroke	56 (80.0)	29 (80.6)	27 (79.4)	
Hemorrhagic stroke	11 (15.7)	5 (13.9)	6 (17.6)	
NIHSS score				
at Admission	2.7 (3.5)	3.5 (3.5)	1.9 (3.4)	0.057
at Discharge	2.3 (3.8)	2.4 (3.1)	2.2 (4.3)	0.768
at 6 month	1.5 (2.9)	1.4 (2.4)	1.6 (3.3)	0.68
mRS				
at Admission				0.193
0, 1	46 (65.7)	21 (58.3)	25 (73.5)	
2	12 (17.1)	6 (16.7)	6 (17.6)	
≥3	12 (17.1)	9 (25.0)	3 (8.8)	
at Discharge				0.384
0, 1	48 (68.6)	22 (61.1)	26 (76.5)	
2	11 (15.7)	7 (19.4)	4 (11.8)	
≥3	11 (15.7)	7 (19.4)	4 (11.8)	
at 6 month				0.848
0, 1	53 (75.7)	26 (72.2)	27 (79.4)	
2	9 (12.9)	5 (13.9)	4 (11.8)	
≥3	8 (11.4)	5 (13.9)	3 (8.8)	
Clinical course				
Preoperative period				**<0.001**
Fluctuation	8 (11.4)	8 (22.2)	0 (0.0)	
Progression	8 (11.4)	8 (22.2)	0 (0.0)	
Postoperative period				**0.003**
Improvement	10 (14.3)	10 (27.8)	0 (0.0)	
Stable	45 (64.3)	18 (50.0)	27 (79.4)	
Fluctuation	13 (18.5)	6 (16.7)	7 (20.6)	
Progression	2 (2.9)	2 (5.5)	0 (0.0)	
Day of hospitalization				
Preoperative period	7.0 (3.8)	8.4 (4.3)	5.6 (2.5)	**0.001**
Postoperative period	9.4 (7.6)	10.5 (10.1)	8.1 (3.1)	0.187
Total	16.4 (9.0)	19.0 (11.6)	13.7 (3.5)	**0.013**
Erythropoietin	68 (97.1)	34 (94.4)	34 (100.0)	0.493
EPO-related complications				
Fever	40 (57.1)	18 (50.0)	22 (64.7)	0.317
Headache	12 (17.1)	4 (11.1)	8 (23.5)	0.289
Surgical site complications	7 (10.0)	1 (2.8)	6 (17.6)	0.052
Lesion extension	3 (4.3)	3 (8.3)	0 (0.0)	**<0.001**
Anesthesia				0.999
Local	67 (95.7)	34 (94.4)	33 (97.1)	
General	3 (4.3)	2 (5.6)	1 (2.9)	

HTN, Hypertension; DM, Diabetes mellitus; TIA, Transient ischemic attack; NIHSS, National Institutes of Health Stroke Scale; mRS, modified Ranking Scale; SD, standard deviation; EPO, Erythropoietin. Bold values means *p*-value < 0.05.

The mean NIHSS score at admission was 2.7, improving to 1.5 at 6 months postoperatively, with significant improvement observed overall (Friedman’s analysis). However, there was no significant difference in the mRS scores between groups at admission, discharge, or 6 months postoperatively (Supplementary Figure 2).

### 3.2 Hemispheric demographics and angiographic characteristics

Among 121 moyamoya hemispheres, 91 underwent MBH. Hemispheres that underwent surgery were more likely to be at an advanced Suzuki stage (*p* = 0.011) and had impaired vascularity preoperatively (*p* < 0.001). Follow-up DSA indicated improvement in vascularity and a shift toward advanced Suzuki stages (Table 2; Supplementary Figure 3).

**Table 2:** Demographics and radiographic characteristics of moyamoya hemispheres.

	All hemispheres (*n* = 121)	Not operated (*n* = 30)	Operated (*n* = 91)	*p*-value
Preoperative Suzuki stage				**0.011**
Stage I-II	17 (14.0)	9 (30.0)	8 (8.8)	
Stage III	26 (21.5)	7 (23.3)	19 (20.9)	
Stage IV	60 (49.6)	8 (26.7)	52 (57.1)	
Stage V	12 (9.9)	4 (13.3)	8 (8.8)	
Stage VI	6 (5.0)	2 (6.7)	4 (4.4)	
Postoperative Suzuki stage				**<0.001**
Stage I-II	12 (9.9)	8 (26.7)	4 (4.4)	
Stage III	10 (8.3)	9 (30.0)	1 (1.1)	
Stage IV	21 (17.4)	6 (20.0)	15 (16.5)	
Stage V	47 (38.8)	4 (13.3)	43 (47.3)	
Stage VI	31 (25.6)	3 (10.0)	28 (30.8)	
Preoperative vascularity				**<0.001**
Impaired	60 (49.6)	4 (13.3)	56 (61.5)	
Not impaired	61 (50.4)	26 (86.7)	35 (38.5)	
Postoperative vascularity				0.999
Impaired	16 (13.2)	4 (13.3)	12 (13.2)	
Not impaired	105 (86.8)	26 (86.7)	79 (86.8)	
Laterality (Left side)	61 (50.4)	17 (56.7)	44 (48.4)	0.562

CTP, CT perfusion; MRP, MR perfusion; SPECT, Single-photon emission computed tomography; RPI, Regional perfusion impairment. Bold values means *p*-value < 0.05.

The hemispheres that underwent surgery also demonstrated more impaired vascularity on preoperative DSA (*p* < 0.001). However, vascularity in these hemispheres had improved on follow-up DSA, and the initial difference between the groups was no longer evident (Table 2).

In the 91 operated hemispheres, early surgical intervention resulted in better outcomes: 72.3% of early group hemispheres achieved Matsushima grade A compared to lower grades in the later group. Additionally, 63.8% of the early group achieved significant reduction (≥50%) of basal moyamoya vessels compared to 27.3% in the later group (Table 3).

**Table 3:** Baseline characteristics and radiographic outcomes of operated hemispheres at follow up angiography.

	All operated hemispheres (*n* = 91)	Early group (*n* = 47)	Later group (*n* = 44)	*p*-value
Suzuki stage				0.133
Stage I-II	8 (8.8)	6 (12.8)	2 (4.5)	
Stage III	19 (20.9)	11 (23.4)	8 (18.2)	
Stage IV	44 (48.4)	27 (57.4)	25 (56.8)	
Stage V	9 (9.9)	1 (2.1)	7 (15.9)	
Stage VI	11 (12.1)	2 (4.3)	2 (4.5)	
Perfusion status (CTP, MRP, SPECT)				**0.001**
Without RPI	7 (7.7)	1 (2.1)	6 (13.6)	
RPI without infarction	62 (68.1)	28 (59.6)	34 (77.3)	
RPI with acute infarction	20 (22.0)	17 (36.2)	3 (6.8)	
RPI with chronic infarction	2 (2.2)	1 (2.1)	1 (2.3)	
Matsushima grade				**0.019**
C	17 (18.7)	6 (12.8)	11 (25.0)	
B	21 (23.1)	7 (14.9)	14 (31.8)	
A	53 (58.2)	34 (72.3)	19 (43.2)	
Basal moyamoya reduction				**0.001**
< 50%	49 (53.8)	17 (36.2)	32 (72.7)	
≥50%	42 (46.2)	30 (63.8)	12 (27.3)	
Regional neovascularization				
Burr hole at frontal bone				**0.042**
Not done	36 (39.6)	19 (40.4)	17 (38.6)	
No replacement	14 (15.4)	3 (6.4)	11 (25.0)	
Balanced	3 (3.3)	1 (2.1)	2 (4.5)	
Replaced	38 (41.8)	24 (51.1)	14 (31.8)	
Burr hole at coronal suture				**0.041**
Not done	10 (11.0)	5 (10.6)	5 (11.4)	
No replacement	15 (16.5)	4 (8.5)	11 (25.0)	
Balanced	12 (13.2)	4 (8.5)	8 (18.2)	
Replaced	54 (59.3)	34 (72.3)	20 (45.5)	
Burr hole at parietal bone				**0.046**
Not done	11 (12.1)	6 (12.8)	5 (11.4)	
No replacement	13 (14.3)	4 (8.5)	9 (20.5)	
Balanced	12 (13.2)	3 (6.4)	9 (20.5)	
Replaced	55 (60.4)	34 (72.3)	21 (47.7)	
Burr hole at posterior parietal				0.088
Not done	65 (71.4)	31 (66.0)	34 (77.3)	
No replacement	9 (9.9)	4 (8.5)	5 (11.4)	
Balanced	6 (6.6)	6 (12.8)	0 (0.0)	
Replaced	11 (12.1)	6 (12.8)	5 (11.4)	

CTP, CT perfusion; MRP, MR perfusion; SPECT, Single-photon emission computed tomography; RPI, Regional perfusion impairment. Bold values means *p*-value < 0.05.

### 3.3 Factors associated with hemispheric neovascularization

In achieving Matsushima grade A, the early group demonstrated significantly improved neovascularization (odds ratio [OR] = 3.44; 95% confidence interval [CI]: 1.46–8.45; *p* < 0.01). However, multivariate analysis showed no significant difference (OR = 2.57; 95% CI: 0.99–6.87; *p* = 0.06). Notably, the presence of RPI with acute infarction was significantly associated with achieving Matsushima grade A in the univariate (OR = 24.00; 95% CI: 3.00–529.73; *p* < 0.01) and multivariate models (OR = 13.28; 95% CI: 1.48–306.91; *p* = 0.04).

Regarding the decrease in basal moyamoya vessels, the early group showed a statistically significant association in the univariate (OR = 4.71; 95% CI: 1.97–11.82; *p* < 0.001) and multivariate analyses (OR = 3.86; 95% CI: 1.52–10.25; *p* < 0.01). Although the presence of RPI with acute infarction was significant in the univariate analysis, this significance was not observed in the multivariate model (Table 4).

**Table 4:** Logistic regression for factors associated with successful revascularization.

	Matsushima grade A (revascularization ≥66%)				Basal moyamoya reduction ≥50%			
	Univariate analysis		Multivariate analysis		Univariate analysis		Multivariate analysis	
	OR [95% CI]	*p-*value	OR [95% CI]	*p-*value	OR [95% CI]	*p-*value	OR [95% CI]	*p-*value
Timing of surgery								
Later group	ref		ref		ref		ref	
Early group	3.44 [1.46–8.45]	**<0.01**	2.57 [0.99–6.87]	0.06	4.71 [1.97–11.82]	**<0.001**	3.86 [1.52–10.25]	**<0.01**
Preoperative vascularity								
Impaired	ref				ref			
Not impaired	0.64 [0.27–1.50]	0.3			0.97 [0.41–2.27]	0.95		
Suzuki stage								
Stage I-II	ref				ref			
Stage III-IV	3.86 [0.86–0.40]	0.08			2.22 [0.50–11.60]	0.3		
Stage V-VI	0.71 [0.13–4.38]	0.7			0.29 [0.04–2.01]	0.2		
Perfusion status (CTP, MRP, SPECT)								
Without RPI	ref		ref		ref		Ref	
RPI without infarction	8.31 [1.31–161.94]	0.06	6.6 [1.00–30.69]	0.09	4.63 [0.73–90.18]	0.17	3.24 [0.47–65.35]	0.3
RPI with acute infarction	24.00 [3.00–529.73]	**<0.01**	13.28 [1.48–306.91]	**0.04**	11.14 [1.50–233.80]	**0.04**	4.76 [0.54–106.39]	0.21
Laterality (Left side)	1.07 [0.46–2.48]	0.87			1.35 [0.59–3.11]	0.48		

CTP, CT perfusion; MRP, MR perfusion; SPECT, Single-photon emission computed tomography; RPI, Regional perfusion impairment; OR, Odds ratio; CI, Confidence interval. Bold values means *p*-value < 0.05.

### 3.4 Factors associated with regional neovascularization

Early surgical intervention was a significant predictor of successful neovascularization in most regions except the posterior parietal region. Impaired vascularity and RPI without infarction were significant predictors in specific regions (Supplementary Table 2).

### 3.5 Survival analysis of adverse events

**Figure 2:**
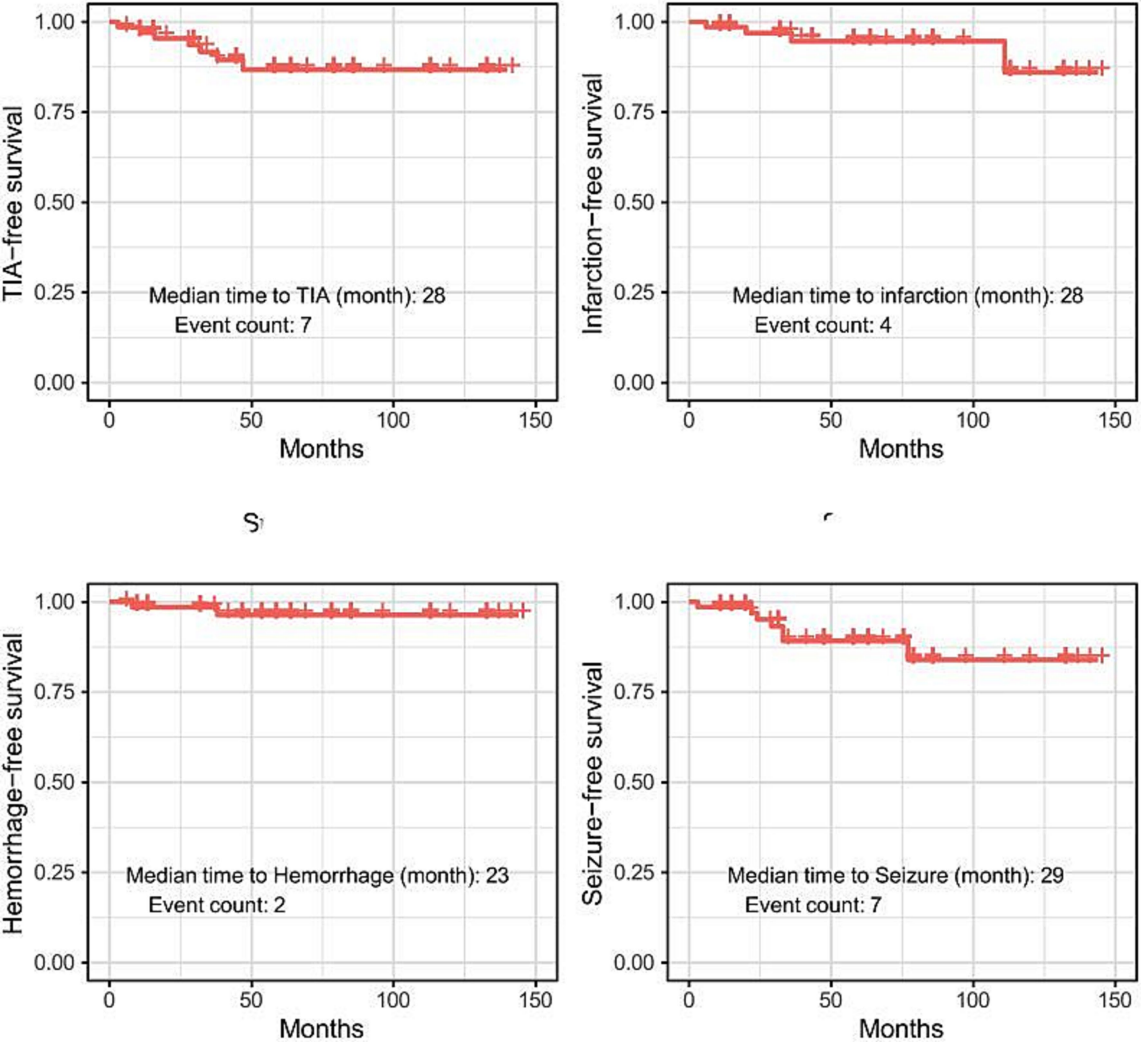
Survival analysis of adverse events. TIA, transient ischemic attack.

We performed survival analysis for each instance of TIA, infarction, hemorrhage, and seizure. Of the 70 patients, TIA occurred in seven individuals, with a median time to TIA of 28 months. Infarction was observed in four patients, with a median time to infarction of 28 months. Hemorrhage occurred in two patients, with a median time to hemorrhage of 23 months. Seizures were observed in seven patients, with a median time to seizure of 29 months. The mean follow-up duration was 56.5 months, with a standard deviation of 37.6 months (Figure 2).

### 3.6 Illustrative cases

#### 3.6.1 Case 1: successful recovery and revascularization following combination therapy in moyamoya-induced infarction

**Figure 3:**
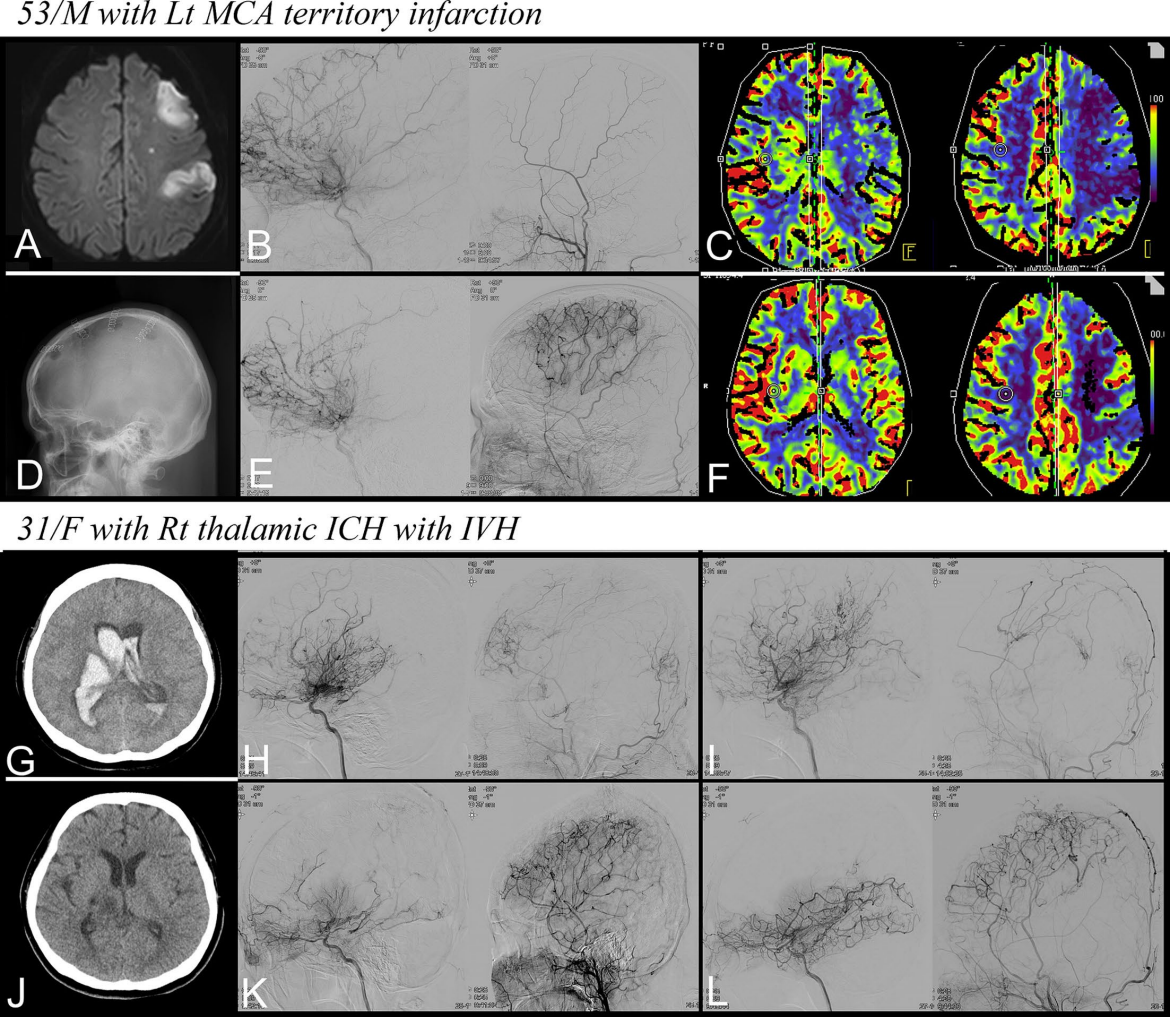
Representative cases. **(A–F)** A 53-year-old man with recent infarction in the left MCA territory. **(A)** Diffusion restriction in the left MCA territory, **(B)** Preoperative DSA for left ICA and ECA demonstrating moyamoya vessels, **(C)** Reduction in CBF on CTP, **(D)** Four multiple burr holes at the left hemisphere, **(E)** Follow-up DSA showing successful revascularization, **(F)** improvement in CBF on follow-up CTP. **(G–L)** A 31-year-old woman with right thalamic ICH with IVH. **(G)** Non-contrast CT at admission, **(H,I)** preoperative DSA for each hemisphere, **(J)** Non-contrast CT at discharge, and **(K,L)** Follow-up DSA showing successful revascularization. MCA, middle cerebral artery; ICA, internal carotid artery; ECA, external carotid artery; DSA, digital subtraction angiography; CBF, cerebral blood flow; CTP, CT perfusion; ICH, intracerebral hemorrhage; IVH, intraventricular hemorrhage; CT, computed tomography.

A 53-year-old man presented with right hemiparesis (Grade 4), hypoesthesia, and dysarthria on admission (NIHSS score: 3, mRS score: 1). Magnetic resonance imaging revealed diffusion restriction in the left MCA territory (Figure 3A). DSA demonstrated moyamoya vessels (Figure 3B) along with a significant reduction in cerebral blood flow (CBF) compared with the contralateral hemisphere (Figure 3C).

The patient’s symptoms worsened during hospitalization, with hemiparesis deteriorating to Grade 3 and the occurrence of aphasia (NIHSS score: 9). Flow-augmentation therapy was administered to stabilize the patient’s hemodynamic condition. The patient underwent combination therapy with MBH and EPO under local anesthesia on the 11th day of hospitalization (Figure 3D). No surgery-related complications were observed, and the patient’s neurological status improved, allowing for participation in rehabilitation programs.

At discharge, 23 days postoperatively, the patient’s NIHSS and mRS scores were 4 and 2, respectively. After a 6-month follow-up, the right hemiparesis continued to improve, with NIHSS and mRS scores of 2 and 1, respectively. Follow-up DSA showed successful revascularization through the burr holes, and the moyamoya vessels were significantly replaced (Figure 3E). CBF also significantly improved at follow-up CTP (Figure 3F). Notably, the patient experienced no adverse events, such as TIA, infarction, hemorrhage, or seizures, throughout the follow-up period of 32 months.

#### 3.6.2 Case 2: successful neovascularization and clinical improvement for patient with hemorrhagic presentation

A 31-year-old woman presented with a stuporous mental state and left hemiparesis (NIHSS score: 15, mRS score: 5). A computed tomography scan at admission revealed a right thalamic hemorrhage with an intraventricular hemorrhage (Figure 3G) leading to the insertion of an emergent extraventricular drain. DSA was performed after treating the increased intracranial pressure and stabilizing the patient’s condition, which revealed the presence of moyamoya vessels bilaterally (Figures 3H,I).

On day 21 of hospitalization, MBH and EPO combination therapy was administered to both hemispheres. Owing to the patient’s dependence on mechanical ventilation, surgery was conducted under general anesthesia with strict hemodynamic monitoring. Four days postoperatively, the patient’s mental status declined again to a state of stupor accompanied by a left-sided eyeball deviation. However, the patient’s mental status improved after administering levetiracetam. The patient’s overall clinical status improved, leading to successful extubation, and the patient could participate in rehabilitation.

Upon discharge, the intracranial hemorrhages had completely resolved (Figure 3J), and the patient’s mRS score was 4; the patient was still bedridden but could communicate clearly. After a 6-month follow-up, her right lower motor strength improved to grade 3, with an mRS score of 3. Follow-up DSA revealed successful revascularization in both hemispheres, and the abnormal moyamoya vessels had significantly regressed (Figures 3K,L). Throughout the 30-month follow-period, the patient experienced no adverse events. Notably, at the last follow-up, the patient’s modified mRS score further improved to 1. She only had a subtle weakness in her left hand but was capable of complete self-care and could start searching for new employment.

## 4 Discussion

In this study, early surgical intervention in patients with moyamoya disease resulted in better angiographic outcomes compared to later intervention. Specifically, early intervention was associated with a higher rate of achieving Matsushima grade A, significant reduction in basal moyamoya vessels, and improved neovascularization in most regions. These findings suggest that early surgical intervention may enhance revascularization and improve neurological outcomes in patients with moyamoya disease or syndrome.

The early group demonstrated significantly better hemispheric neovascularization outcomes, with 72.3% achieving Matsushima grade A compared to lower grades in the later group. Additionally, 63.8% of early group patients showed a significant reduction (≥50%) in basal moyamoya vessels, compared to only 27.3% in the later group. The presence of RPI with acute infarction was also a significant predictor of achieving Matsushima grade A, indicating that targeting patients with acute ischemic events may be particularly beneficial for successful neovascularization.

Studies have investigated the MBH as the sole method of indirect bypass surgery for patients with MMD or MMS (19–22). In 1996, Kawaguchi et al. performed MBH in 10 adults and reported favorable outcomes (23). The study highlighted that MBH can be performed without craniotomy and general anesthesia, avoiding perioperative hypotension and hypercapnia. They also showed that angiogenesis occurs efficiently in areas of decreased CBF on SPECT scans. Saint Rose et al. performed MBH in 64 pediatric patients and presented the long-term follow-up results of their study (22, 24). The surgery was performed under general anesthesia, and a higher number of burr holes were applied. The MBH procedure introduced in our study is similar to the method described by Kawaguchi et al.

The safety of bypass surgery in patients with acute stroke episode of MMD or MMS has long been a debatable issue. Safety concerns arise primarily owing to hypotension during general anesthesia, hyperventilation during awakening, and intraoperative bleeding leading to the possibility of postoperative stroke (7–12). Recent studies examining the appropriate timing of surgery for patients with MMD or MMS following an acute stroke episode show that 15–22% of patients who underwent bypass surgery within 90 days of an acute stroke episode experienced postoperative strokes, and 17–33% experienced neurological deterioration (13, 14). These findings might suggest that early surgery is riskier; however, it is crucial to recognize the heterogeneity in surgical methods between early and delayed surgery groups in previous studies. This variability makes it challenging to conclude that early surgical intervention carries inherently greater risks. By comparing groups treated with the same MBH technique, our study offers enhanced validity and consistency in determining the true impact of surgery timing. Unlike previous studies, we defined the early group as patients who underwent MBH less than 30 days from the most recent neurological event 30 days, potentially targeting more unstable patients. The rates of postoperative fluctuation and progression were nearly identical in the early and later groups. Notably, only 5.5% in the early group experienced neurological worsening, significantly lower than the 17–33% rate reported in previous studies (13, 14). Furthermore, lesion extension occurred in 8.3% patients in the early group, with two cases being asymptomatic; this is also lower than the 15–22% rate reported previously (13, 14). In fact, 27.8% of patients in our early group showed neurological improvement. Concurrent medication therapy may have played a role; however, this suggests that some patients who experience an acute stroke episode can achieve clinical improvement early through MBH.

Previous animal studies have demonstrated that erythropoietin (EPO) induces neuroprotective and angiogenic effects. Notably, combination therapy involving EPO in animal models has been shown to facilitate arteriogenesis and enhance the maturation of newly developed vessels (17). Additionally, the benefits of EPO were examined in a human randomized controlled trial involving stroke patients with cerebral hypoperfusion. This study compared outcomes between patients received MBH alone and those underwent combination therapy with EPO, showing significantly improved angiographic outcome in combination therapy group (16). Moreover, as MBH barely disrupts anatomical structures, it allows for additional surgeries if collateral formation is insufficient. Therefore, employing MBH and EPO therapy first in patients with acute stroke episode, followed by considering additional bypass surgery during a stable phase, could be an excellent treatment strategy.

Furthermore, we divided the regions where burr holes were performed to investigate the factors associated with the neovascularization in detail. Impaired vascularity and perfusion demonstrated significant associations with neovascularization, which was particularly evident in the regions supplied by the ACA and MCA. Notably, in regions with perfusion impairment, neovascularization was less pronounced in areas affected by chronic infarction and atrophy. In summary, there was a tendency for better neovascularization when the vascularity and perfusion status were not optimal and when the surgery was performed early. However, this pattern was not observed in the posterior parietal region. This finding indicates that the angiogenic demand of the salvageable penumbra influences neovascularization.

Furthermore, as illustrated in Case 2, excellent neovascularization was observed even in patients with hemorrhage. According to a study based on data from the Japan Adult Moyamoya Trial, the reduction in rebleeding after bypass surgery in patients with hemorrhage was more effective when SPECT showed a lower cortical hemodynamic grade (25, 26). This suggests that a subgroup of patients with hemorrhage benefits more from bypass surgery. Therefore, we speculate that the timing of surgery might be a critical factor for neovascularization and may contribute to the reduction of abnormal vessels, even in patients with hemorrhagic stroke.

Finally, we conducted a follow-up of patients for an average of 56.5 months. Among the 70 patients, only 6 experienced infarction and hemorrhage. To our knowledge, this is the largest and longest follow-up study involving adult patients who underwent MBH as the sole method of indirect bypass, which adds to the promising evidence supporting the feasibility and potential benefits of MBH and EPO combination therapy for managing patients with MMD or MMS.

This study had some limitations. First, this was a single-center, retrospective study. Second, this study was limited by the lack of a control group. Therefore, definitively concluding the superiority of combination therapy with MBH and EPO over other surgical options is challenging. Third, in this study, when dealing with basal moyamoya vessels, we did not analyze periventricular anastomosis, a well-known risk factor for hemorrhagic moyamoya (27, 28). Instead, we arbitrarily set a criterion of more than 50% reduction in moyamoya vessels for our analysis. Research on periventricular anastomosis is expected to be an important topic in the future. Fourth, we did not compare the MBH and EPO combination therapy group with the MBH only group, thus we were unable to prove that EPO combination is essential for collateral formation. Finally, long-term and systematic observation and prognostic evaluation of the treated patient groups are necessary.

In conclusion, the MBH and EPO combination therapy is an effective, minimally invasive treatment option for patients with an acute stroke episode of MMD or MMS. Owing to the minimally invasive nature of MBH, surgical intervention in the early period could be a safe treatment option compared to later intervention and may even be beneficial regarding angiographic outcomes. We suggest that performing that MBH and EPO therapy first in patients with an acute stroke episode, followed by considering additional bypass surgery during a stable phase, could be a safe and effective treatment strategy.

## Data Availability

The datasets presented in this article are not readily available because the datasets analyzed in this study are not publicly available due to privacy concerns. Requests to access the datasets should be directed to strokomong14@gmail.com.
