# Supplementary material for: Multiple burr hole and erythropoietin combination therapy: optimal early surgical intervention for patients with acute stroke episode of moyamoya disease or moyamoya syndrome
Source: Front Neurol. 2024 Dec 23;15:1479379. doi: 10.3389/fneur.2024.1479379 (PMC11700813; doi:10.3389/fneur.2024.1479379)
Supplement: Supplementary file 1 [file Data_Sheet_1.pdf]

Supplementary Material

Supplementary Figures and Tables

Supplementary Figure 1

1A: Example of “Impaired” vascularity on lateral DSA view.

1B: Example of “Not impaired” vascularity on lateral DSA view.

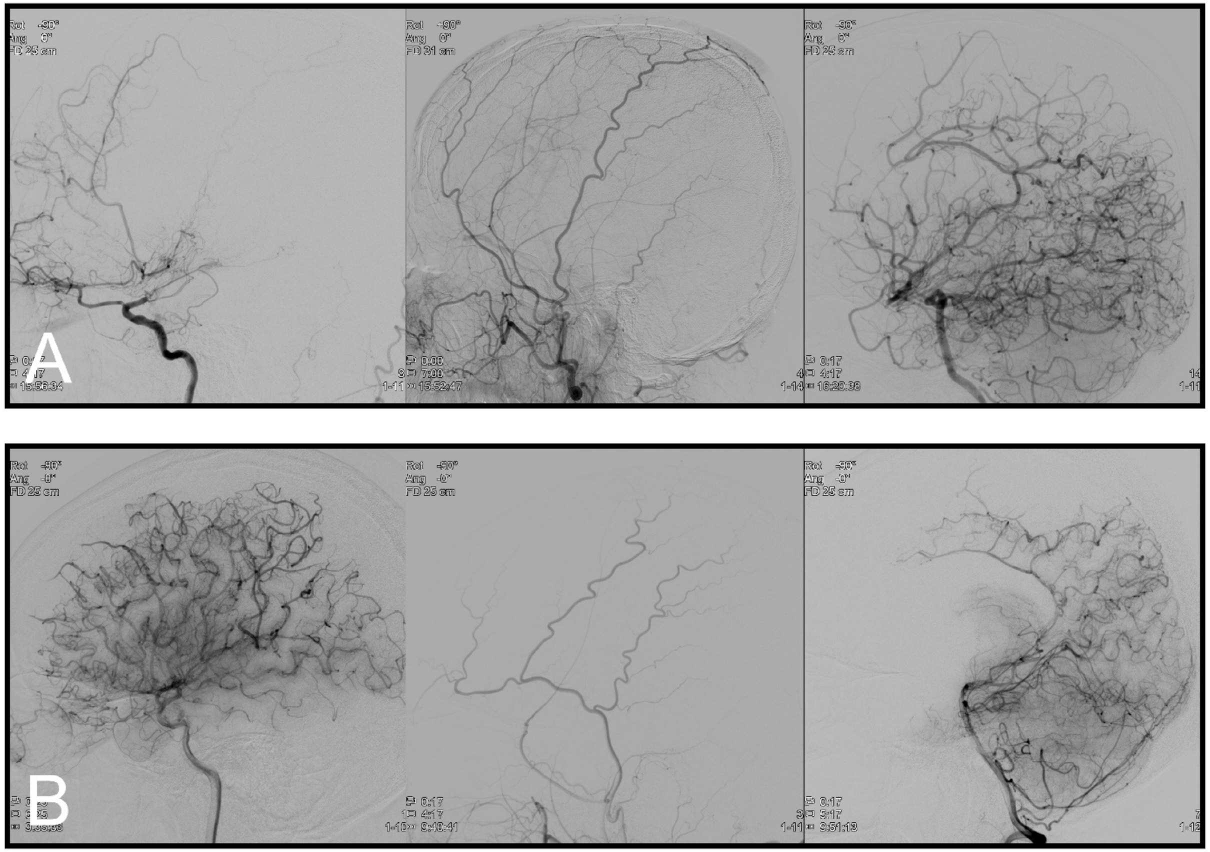

DSA: Digital subtraction angiography

Supplementary Figure 2:

Violin plots for NIHSS scores at different timepoints

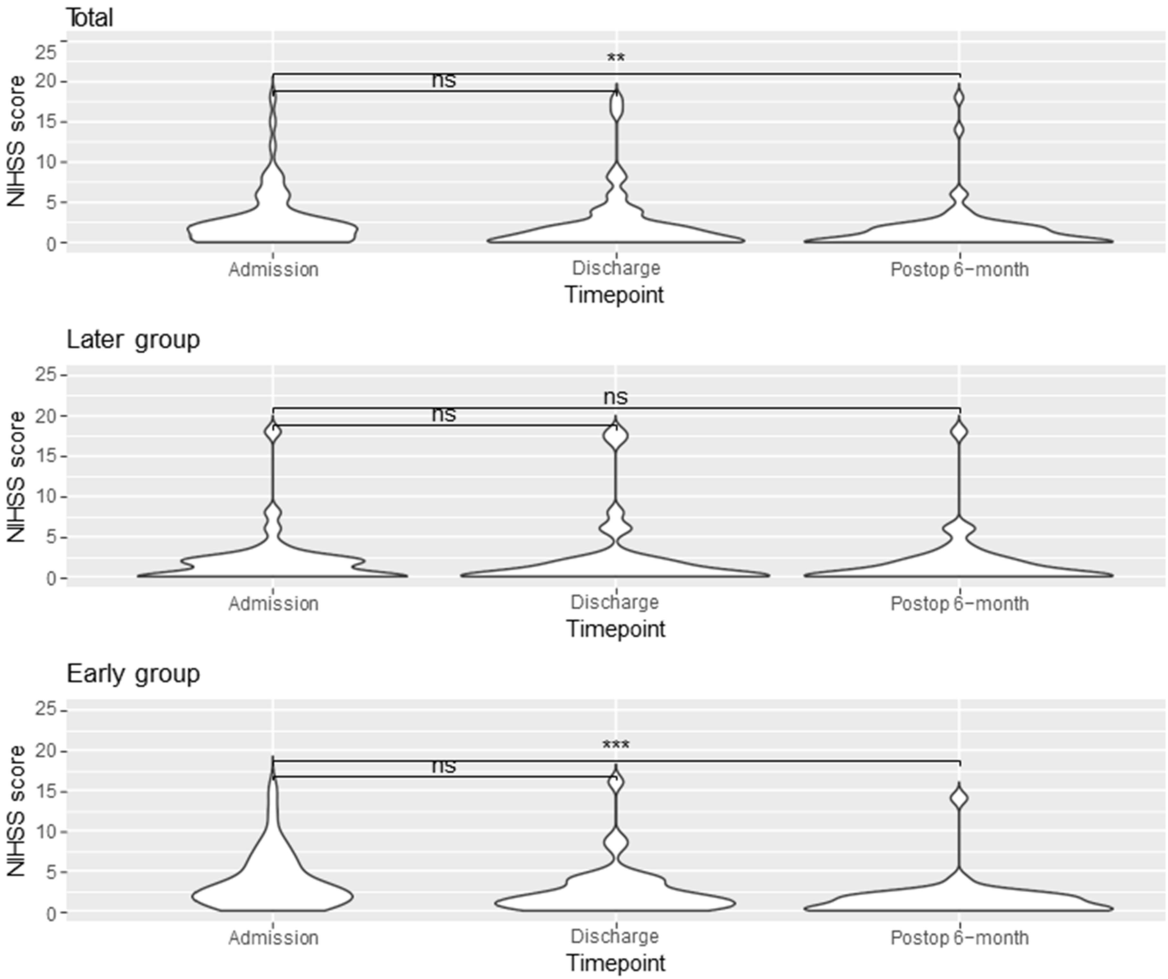

NIHSS: National Institutes of Health Stroke Scale, ns: not statistically significant, **: p-value < 0.01, ***: p-value < 0.001

Supplementary Figure 3

Violin plots for Suzuki stages at different timepoints

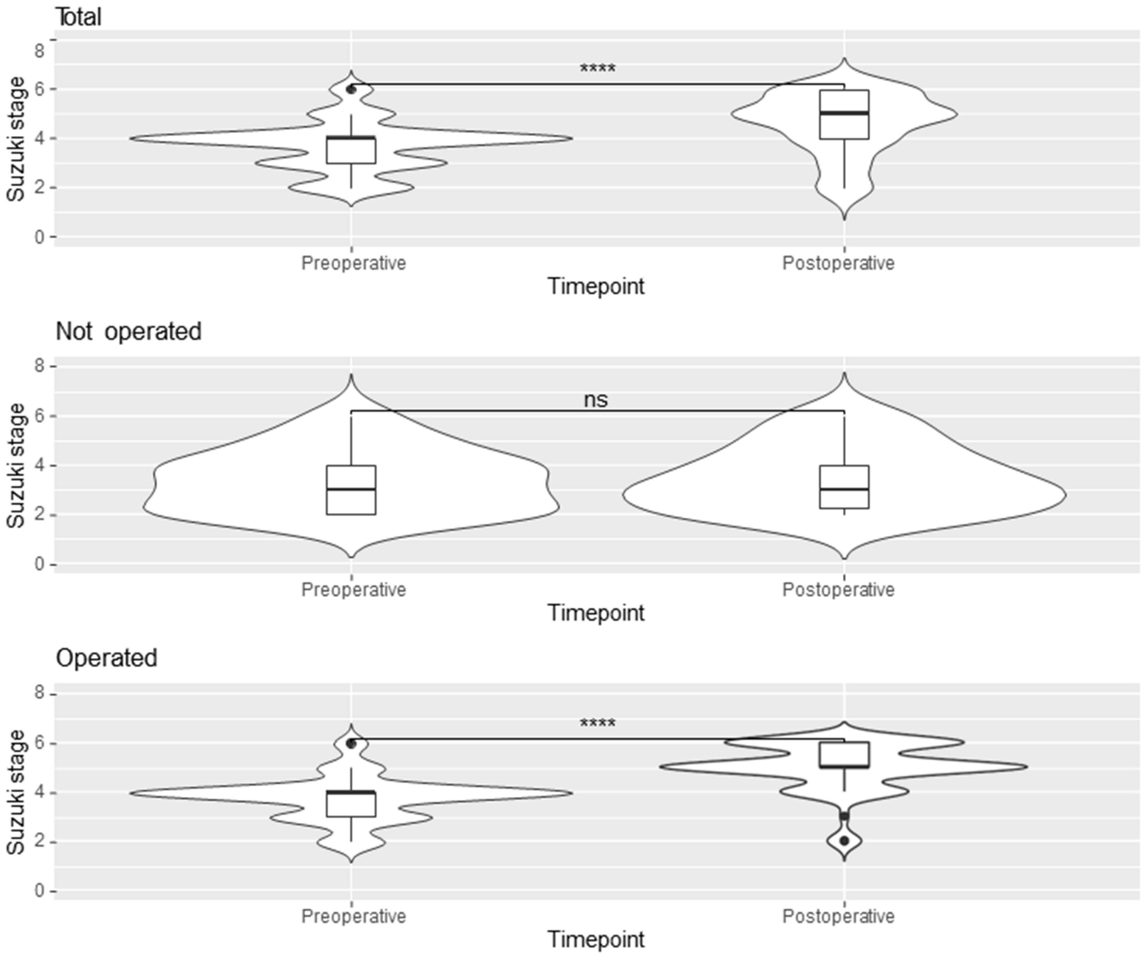

ns: not statistically significant, **: p-value < 0.01, ***: p-value < 0.001

Supplementary Table 1

Clinical details of patients showing fluctuations or progression in the postoperative period

Post operative clinical worsening	Age/sex	Presentation	MRI lesion	Pre operative clinical course	Detailed clinical course	Discharge mRS	6m mRS
Lesion extension 1<br>Progression 1	52/F	Right hemiplegia<br>upper and lower G1<br>NIHSS 9	Acute,<br>Lt MCA<br>dotty infarct	Progression	Seven days after the procedure, the patient exhib ited mixed transcortical aphasia, and an extensio n of the lesion was identified at the corpus callo sum on MR-DWI. However, this lesion extension showed improvement with flow augmentation th erapy.	4	2
Lesion extension 2<br>Progression 2	42/F	Clinical TIA<br>Motor weakness, dysarthria<br>within 2 weeks	Acute,<br>Lt MCA<br>dotty infarct	Fluctuation	During the first two days after the procedure, the patient experienced transient aphasia, which subs equently showed improvement. Upon routine CT scan before discharge, an extension of the lesion is observed in the left frontoparietal area.	2	2
Lesion extension 3<br>Fluctuation 1	57/M	Hemispheric symptoms<br>NIHSS 22	Acute,<br>Lt MCA<br>Borderzone infarct	Progression	Following the combination therapy, the patient's neurological status showed improvement to an NI HSS score of 14 after 2 days. However, a follow -up CT scan revealed an extension of the hemisp heric lesion.	4	4
Fluctuation 2	39/F	Clinical TIA<br>Lt hand hypoesthesia, Headache<br>At admission, no clinical symptoms	TIA<br>without any parenchymal abnormality	Stable	Seven days after the procedure, the patient experi enced temporary weakness in the distal upper mo tor (graded as G4). This weakness was successful ly resolved after receiving hydration and dual ant iplatelet therapy. Additionally, the MRI did not s how any abnormalities.	1	1
Fluctuation 3	61/F	Aphasia, Right side weakness,<br>one month before admission<br>without clinical aggravation	Lt ACA<br>dotty infarct	Stable	Five days after the procedure, the patient experie nced a brief episode of transient hypoesthesia in her right finger, lasting for approximately 15 min utes.<br>This hypoesthesia resolved on its own without th e need for intervention.	0	1
Fluctuation 4	49/F	IVH treated with EVD,<br>6month before admission.<br>At admission, no clinical symptoms	No parenchymal abnormality	Stable	Five days after the procedure, the patient experie nced temporary weakness in her left fingers (G 4). However, this weakness improved after receiv ing cilostazol.<br>The MRI did not show any abnormal signals.	1	1

Fluctuation 5	31/F	Right thalamic ICH c IVH treated with EVD, Stupor mentality at admission	Not taken	Stable, Mental status was improved	Four days after the procedure, the patient's menta l status declined again to a state of stupor, acco mpanied by left-sided eyeball deviation. Howeve r, her mental status improved after the administra tion of levetiracetam. The patient's general clinica l status improved, leading to successful extubatio n and successful rehabilitation.	4	3
Fluctuation 6	38/F	Clinical TIA Lt leg hypoesthesia No focal neurologic deficit at admission	No parenchymal abnormality	Stable	Six days after the procedure, the patient experien ced transient hypoesthesia in the left arm, which resolved on its own. Additionally, CT angiograph y did not reveal any abnormalities.	0	0
Fluctuation 7	25/F	Clinical TIA Lt hand hypoesthesia, Headache No focal neurologic deficit at admission	White matter signal change, Both internal border zone	Stable	Four days after the procedure, the patient experie nced mild hypoesthesia in the left arm and leg. T his symptom was resolved after administration of cilostazol.	0	0
Fluctuation 8	70/F	Lt hemiparesis, Lt homonymous hemianopsia due to infarction, 4 years before admission. Disease status was stable at admission	Rt PCA infarct, both basal ganglia dotty infarct	Stable	Three days after the procedure, the patient develo ped a fever and became abulic. However, her sy mptoms showed improvement after receiving flui d augmentation therapy and antipyretic medicatio ns.	5	3
Fluctuation 9	37/M	Dysarthria NIHSS 1	Acute, Lt MCA scattered infarct	Progression Right homonymous hemianopsia and Rt hemiparesis (upper, G4) with hypoesthesia was developed, lesion extension was seen at Lt MCA-PCA, watershed area on MR-DWI	Six days after the procedure, the patient develope d additional right-sided motor weakness (graded as G4) in the lower extremity. However, this wea kness showed improvement with fluid augmentati on and induced hypertension therapy. There were no signs of additional acute infarction on MR-D WI. Other symptoms that worsened just before th e procedure showed improvement. By the time of discharge, only subtle hypoesthesia remained.	1	1
Fluctuation 10	27/F	Clinical TIA Rt hand, leg weakness No focal neurologic deficit admission	No parenchymal abnormality	Stable	Right after the procedure, the patient experienced a focal motor onset seizure that lasted for 30 se conds. Fortunately, the seizure resolved spontaneo usly.	0	0
Fluctuation 11	46/F	Clinical TIA	Acute, Rt MCA dotty infarct	Progression	Immediately after the procedure, the patient repor ted transient hypoesthesia in the left hand, which resolved on its own. By the time of discharge, t	2	1

		Lt hand motor weakness		Motor weakness reoccured before the procedure	he motor weakness in the left hand had fully rec overed.		
Fluctuation 12	35/F	Cortical TIA symptoms	Acute, Lt borderzone dotty infarct	Stable	Three days post-procedure, the patient encountere d tingling in the right hand and distal weakness with a grade of 4. These symptoms resolved with in a span of two hours.	0	0
Fluctuation 13	72/F	Motor weakness NIHSS 0	Acute, Lt MCA posterior borderzone dotty infarct	Stable	Two days after receiving combination therapy, th e patient presented with distal handgrip weakness (graded as G4). However, this condition was suc cessfully resolved with flow augmentation therap y.	2	0

F: Female, M: Male, Lt: Left, Rt: Right G: Motor grade, NIHSS: National Institutes of Health Stroke Scale, TIA: Transient ischemic attack, ACA: Anterior cerebral artery, MCA: Middle cerebral artery, PCA: Posterior cerebral artery, MR-DWI: Diffusion waited image on Magnetic resonance imaging, CT: Computed tomography, EVD: External ventricular drainage, ICH: Intracerebral hemorrhage, IVH: Intraventricular hemorrhage

Supplementary Table 2:

Logistic regression for factors associated with successful regional neovascularization

	Frontal bone				Coronal suture			
	Univariate analysis		Multivariate analysis		Univariate analysis		Multivariate analysis	
	OR [95% CI]	*p*	OR [95% CI]	*p*	OR [95% CI]	*p*	OR [95% CI]	*p*
Vascularity (DSA)								
Frontal, n								
Not impaired	ref		ref		ref			
Impaired	3.5 [1.67 - 7.54]	**<0.01**	1.39 [0.29 - 6.97]	0.68	1.83 [0.72 - 4.72]	0.21		
Coronal suture, n								
Not impaired	ref				ref		ref	
Impaired	2.42 [0.65 - 8.92]	0.18			3.44 [1.21 - 10.08]	**0.02**	5.35 [1.11 - 29.35]	**0.04**
Parietal, n								
Not impaired	ref				ref		ref	
Impaired	7.56 [1.94 - 34.12]	**<0.01**			6.4 [2.12 - 21.27]	**<0.01**	8.05 [1.53 - 55.54]	**0.02**
Posterior parietal, n								
Not impaired	ref				ref			
Impaired	0.59 [0.10 - 1.89]	0.44			0.72 [0.26 - 1.68]	0.48		
Perfusion status (CTP, MRP, SPECT)								
ACA, n								
Without RPI	ref		ref		ref		ref	
RPI without infarction	4.09 [1.08 - 16.97]	**0.04**	5.72 [1.07 - 41.69]	0.06	5.42 [1.81 - 17.62]	**<0.01**	6.05 [1.38 - 31.31]	**0.02**
RPI with acute infarction								
RPI with chronic infarction					4.64 [0.78 - 38.86]	0.11	2.76 [0.30 - 33.24]	0.38
MCA, n								
Without RPI	ref		ref		ref		ref	
RPI without infarction	7.43 [1.82 - 35.47]	**<0.01**	5.33 [1.01 - 35.42]	0.06	4.92 [1.47 - 18.33]	**0.01**	6.03 [1.21 - 35.21]	**0.03**
RPI with acute infarction	14 [1.68 - 314.47]	**0.03**	3.19 [0.23 - 85.74]	0.41	12.6 [1.60 - 273.73]	**0.04**	2.03 [0.15 - 54.05]	0.61
RPI with chronic infarction	2 [0.07 - 60.50]	0.65	2.01 [0.06 - 71.58]	0.67	0.9 [0.04 - 11.95]	0.94	2.37 [0.06 - 75.92]	0.62
PCA, n								
Without RPI	ref				ref			
RPI without infarction	1.17 [0.28 - 6.05]	0.84			2.51 [0.70 - 11.94]	0.19		
RPI with acute infarction					3.14 [0.47 - 62.34]	0.31		
RPI with chronic infarction					1.26 [0.11 - 28.00]	0.86		
Timing of surgery, n								
Later group	ref		ref		ref		ref	
Early group	5.57 [1.62 - 22.95]	**<0.01**	6.22 [1.27 - 40.40]	**0.03**	4.04 [1.54 - 11.41]	**<0.01**	5.82 [1.40 - 30.26]	**0.02**

	Parietal bone				Posterior parietal bone			
	Univariate analysis		Multivariate analysis		Univariate analysis		Multivariate analysis	
	OR [95% CI]	*p*	OR [95% CI]	*p*	OR [95% CI]	*p*	OR [95% CI]	*p*
Vascularity (DSA)								
Frontal, n								
Not impaired	ref				ref			
Impaired	1.62 [0.62 - 4.24]	0.33			1.8 [0.38 - 9.12]	0.46		
Coronal suture, n								
Not impaired	ref		ref		ref			
Impaired	5.54 [1.96 - 16.59]	**<0.01**	6.44. [1.65 - 29.07]	**<0.01**	5.14 [0.93 - 41.95]	**0.08**		
Parietal, n								
Not impaired	ref		ref		ref			
Impaired	7.86 [2.44 - 28.64]	**<0.001**	5.93 [1.31 - 31.29]	**0.03**	2.25 [0.38 - 18.57]	0.4	1.65 [0.24 - 14.76]	0.62
Posterior parietal, n								
Not impaired	ref				ref			
Impaired	0.58 [0.18 - 1.42]	0.28			0.21 [0.03 - 0.90]	**0.07**	0.2 [0.03 - 0.98]2	**0.08**
Perfusion status (CTP, MRP, SPECT)								
ACA, n								
Without RPI	ref				ref			
RPI without infarction	7.2 [2.37 - 23.97]	**<0.001**			2.62 [0.43 - 22.15]	0.32		
RPI with acute infarction								
RPI with chronic infarction	16 [2.29 - 329.08]	**0.02**						
MCA, n								
Without RPI	ref		ref					
RPI without infarction	3.76 [1.05 - 14.40]	**0.04**	5.24 [1.17 - 25.99]	**0.03**				
RPI with acute infarction	8.4 [1.00 - 186.94]	**0.08**	2.94 [0.24 - 75.92]	0.43				
RPI with chronic infarction	1.4 [0.05 - 41.55]	0.83	3.35 [0.09 - 148.36]	0.49				
PCA, n								
Without RPI	ref				ref			
RPI without infarction	1.45 [0.43 - 5.80]	0.57			1.35 [0.20 - 8.86]	0.75		
RPI with acute infarction	2.64 [0.39 - 52.50]	0.39			6.75 [0.64 - 162.80]	0.14		
RPI with chronic infarction	1.58 [0.19 - 33.22]	0.7						
Timing of surgery, n								
Later group	ref		ref		ref			
Early group	4.16 [1.54 - 12.31]	**<0.01**	5.6 [1.56 - 24.86]	**0.01**	0.6 [0.12 - 3.00]	0.53		

DSA: Digital subtraction angiography, CTP: CT perfusion, MRP: MR perfusion, SPECT: Single-photon emission computed tomography, ACA: Anterior cerebral artery, MCA: Middle cerebral artery, PCA: Posterior cerebral artery, RPI: Regional perfusion impairment, OR: Odds ratio, CI: Confidence interval, *p*: p-value
